# Martian outflow channels: How did their source aquifers form, and why did they drain so rapidly?

**DOI:** 10.1038/srep13404

**Published:** 2015-09-08

**Authors:** J. Alexis P. Rodriguez, Jeffrey S. Kargel, Victor R. Baker, Virginia C. Gulick, Daniel C. Berman, Alberto G. Fairén, Rogelio Linares, Mario Zarroca, Jianguo Yan, Hideaki Miyamoto, Natalie Glines

**Affiliations:** 1 Planetary Science Institute, 1700 East Fort Lowell Road, Suite 106, Tucson, AZ 85719-2395, USA; 2 NASA Ames Research Center, Mail Stop 239-20, Moffett Field, CA 94035, USA; 3 Department of Hydrology & Water Resources, University of Arizona, Tucson, AZ 85721, USA; 4 SETI Institute, 189 Bernardo Avenue, Mountain View, CA 94043; 5 Centro de Astrobiología, M-108 km 4, 28850 Madrid, Spain; 6 Department of Astronomy, Cornell University, Ithaca 14850 NY, USA; 7 External Geodynamics and Hydrogeology Group, Department of Geology, Autonomous University of Barcelona, 08193 Bellaterra, Barcelona, Spain; 8 State Key Laboratory of Information Engineering in Surveying, Mapping and Remote Sensing, Wuhan University, Wuhan, 430070, China; 9 The University Museum, University of Tokyo, 113-0033, Japan

## Abstract

Catastrophic floods generated ~3.2 Ga by rapid groundwater evacuation scoured the Solar System’s most voluminous channels, the southern circum-Chryse outflow channels. Based on Viking Orbiter data analysis, it was hypothesized that these outflows emanated from a global Hesperian cryosphere-confined aquifer that was infused by south polar meltwater infiltration into the planet’s upper crust. In this model, the outflow channels formed along zones of superlithostatic pressure generated by pronounced elevation differences around the Highland-Lowland Dichotomy Boundary. However, the restricted geographic location of the channels indicates that these conditions were not uniform Boundary. Furthermore, some outflow channel sources are too high to have been fed by south polar basal melting. Using more recent mission data, we argue that during the Late Noachian fluvial and glacial sediments were deposited into a clastic wedge within a paleo-basin located in the southern circum-Chryse region, which was then completely submerged under a primordial northern plains ocean. Subsequent Late Hesperian outflow channels were sourced from within these geologic materials and formed by gigantic groundwater outbursts driven by an elevated hydraulic head from the Valles Marineris region. Thus, our findings link the formation of the southern circum-Chryse outflow channels to ancient marine, glacial, and fluvial erosion and sedimentation.

At the end of the Noachian Period Mars experienced an abrupt transition into a climate dominated by extremely cold and dry conditions, which resulted in the subsequent confinement of the planet’s hydrosphere in highly pressurized aquifers beneath thick upper crustal permafrost materials^1^,^2^. A popular model proposes that the vast Noachian surface water systems were cold-trapped in the planet’s south polar region to form an ice cap, which gradually infiltrated into a global mega-regolith as a consequence of pressure-induced basal melting^1^,^2^. The catastrophic floods that excavated the Late Hesperian southern circum-Chryse outflow channels^3^ would have occurred in zones where the elevation difference between the Martian south polar region and lower terrains generated superlithostatic pressures^1^,^2^.

In order to explain the relative localized occurrence^4^,^5^ of catastrophic-flood-formed Martian outflow channels, it was hypothesized that the causative flooding emanated from a compartmentalized global hydrosphere that was also contained within a planetwide megaregolith^6^. In this model, the existence of high elevation outflow channels in the circum-Chryse region^7^,^8^ can be explained by the presence of an aquifer system that extended from the Tharsis Montes to the outflow channel head source regions^9^. It was subsequently discovered that rather than possessing global megaregolith, much of the Martian upper crust appears to be dominated by Noachian sedimentary deposits of great stratigraphic thicknesses that both infill and bury numerous impact craters^10^,^11^. Hence, it was proposed that, instead of a megaregolith-trapped hydrosphere^1^,^2^,^6^, significant portions of these outflow-channel-source (OFCS) aquifers might have consisted of water-ice contained within buried impact craters and impact-fractured rocks^12^,^13^. However, because the sedimentary deposits are distributed globally on Mars, the unique conditions leading to the formation of the southern circum-Chryse outflow channel source regions are still poorly understood. Here, we propose a new geologic model linking the development of OFCS aquifers within the highlands of southern circum-Chryse (Fig. 1a) to a stage of Late Noachian large-scale regional marine sedimentation.

## Results and interpretative synthesis

The long-held view is that regional groundwater outbursts led to the formation of extensive chaotic terrains (collapsed upper crustal materials) that are the sources of the southern circum-Chryse outflow channels^5^,^14^,^15^,^16^,^17^,^18^,^19^,^20^. More recently, zones of surface subsidence have been recognized as including much more extensive areas of adjacent highlands^12^,^13^,^21^. Consequently, the distribution of regional highland surfaces modified by collapse and subsidence were proposed to demark the approximate extent of the OFCS aquifers^12^,^13^.

**Figure 1:**
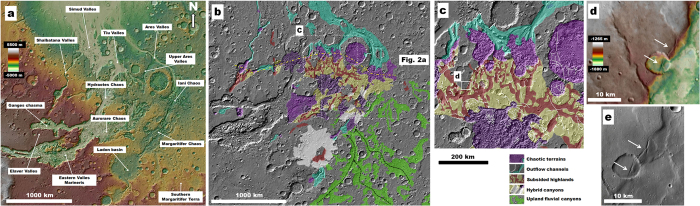
(**a**) Context topographic view of southern circum-Chryse and eastern Valles Marineris showing cited geographic features (MOLA DEM, 460 m/pixel centered at 3.18° S; 332.10° E). (**b**) Geomorphic map of southern circum-Chryse showing the distribution of Noachian fluvial canyons, subsided surfaces, chaotic terrains and outflow channels. Black arrows show the inferred directions of surface flows along the upland canyons. Dashed yellow line traces an equatorial belt of chaotic terrains. (**c**) Close-up view on panel b shows the most extensive zone of subsidence in southern circum-Chryse, including the distribution of fault systems (white lines) in the region. (**d,e**) Close-up views on a subsided valley that exhibit faulted slope breaks (white arrows). We produced the maps in this figure using Esri’s ArcGIS geographic information system.

These chaotic terrains have been mapped in detail by previous investigators^4^,^16^,^22^, and here we present the first map showing the full extent of highland subsidence in this region of the planet (Fig. 1b,c). We show that these previously unmapped subsidence zones modify large areas of the highlands and connect eastern Valles Marineris to the initiation zones of numerous outflow channels (Fig. 1b). The subsided terrains include regionally extensive networks of broad valleys (e.g., Fig. 1b,c) that are characterized by faulted margins (Fig. 1c–e) and abrupt breaks-in-slope (Fig. 1c–e) that deform pre-existing landforms (e.g., Fig. 1d,e). These formational structures have been interpreted as synclinal and monoclinal folds and associated ruptures developed by subsidence over caverns that formed by the removal of ice, water, and fluidized sediment^12^,^13^.

**Figure 2:**
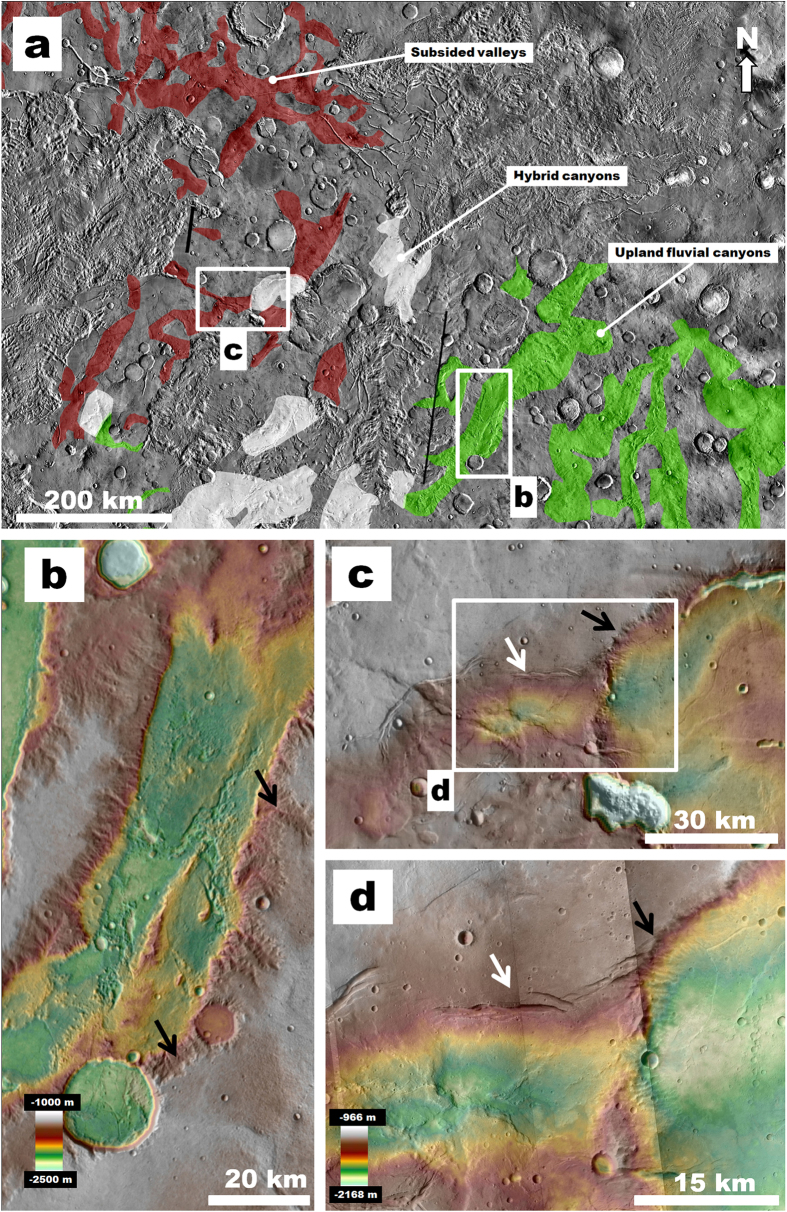
(**a**) Map showing the distribution of hybrid canyons in terrains located along the boundary between upland fluvial canyons and subsided valleys (context and location in Fig. 1b). (**b**) Topographic view of an upland fluvial canyon, which shows margins densely marked by small valleys/channels (black arrows). (**c,d**) Topographic views of a subsided valley (white arrow) that extends eastward to join a hybrid canyon that exhibits a margin dissected by small valleys/channels adjoined by subsidence related fractures (black arrow). We produced the map in this figure using Esri’s ArcGIS geographic information system.

The southern Margaritifer Terra highlands are extensively dissected by vast canyon systems (Fig. 1b and 2a), which exhibit flanks densely marked by channels (e.g., Fig. 2b)^4^. The formation of these canyons is thought to have been dominated by Middle and Late Noachian fluvial erosion^4^. Late Noachian equatorial glaciers may have also contributed to their development^23^. Within the study region, these canyons converge northwestward into the highlands containing the subsided terrains (Fig. 1a,b). Although the contact between these two terrain types is disrupted by the Margaritifer and Iani chaotic terrains, we have identified a relatively narrow stretch of plateau materials that includes a subset of canyons that exhibit transitional morphologies (Fig. 2a). These hybrid canyons include scarp margins marked by dense channel systems and extensional fractures generated by subsidence (Fig. 2c,d), thus offering a rare insight into the regional upper stratigraphy of the outflow channel source region, consistent with the existence of buried canyons underlying the subsided valleys. Similar hybrid morphologies are observable along the flanks of Ladon basin (Fig. 1a), which constitutes large impact basin that partly captured drainage from the upland fluvial canyons (Fig. 1b).

**Figure 3:**
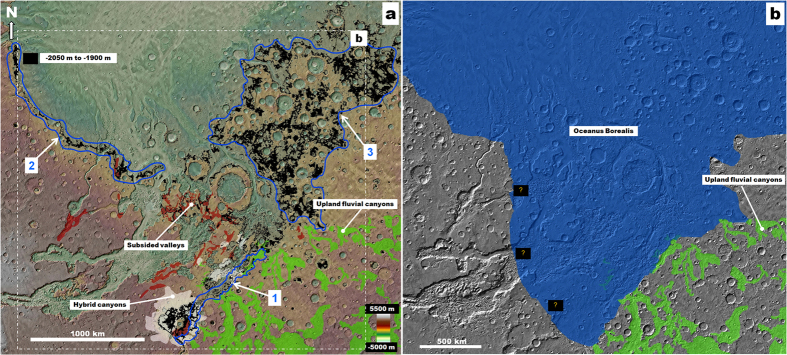
(**a**) Topographic view of circum-Chryse and western Arabia Terra showing the distribution of upland fluvial canyons (green), hybrid canyons (white) and subsided valleys (red). The black areas mark elevations ranging from −2050 to −1900, which mark the contact between the upland fluvial canyons and the subsided terrains (1), the dichotomy boundary west of the outflow channels (2), and the inter-crater plains of western Arabia Terra (3). (**b**) Reconstruction of coastal line at approximately −1900 m during the proposed stage of regional Late Noachian sedimentation. Question marks show the locations of uncertain paleoshoreline stretches. We produced the maps in this figure using Esri’s ArcGIS geographic information system.

The terrain contact between the upland and hybrid canyons is defined by elevations ranging between −2050 m and −1900 m (Fig. 3a, label 1), which also mark a section of the dichotomy boundary to the west (Fig. 3a, label 2). The dichotomy boundary corresponds approximately to the margins of water bodies (“Oceanus Borealis”^24^) that episodically covered the northern plains, most extensively during the Late Noachian^24^,^25^,^26^,^27^,^28^ and Late Hesperian^25^,^26^,^27^,^28^,^29^. The mean elevation of the Late Noachian ocean shoreline, which is the one of relevance to this study, has been estimated to be approximately −1680 m^24^. The elevation range also characterizes the inter-crater plains surfaces in western Arabia Terra (Fig. 3a, label 3), a region of the planet which has been interpreted as a frozen, buried remnant of the Late Noachian ocean^30^, where localized desiccation of the regional hydrosphere generated the widespread evaporite deposits detected by the Opportunity rover^31^.

**Figure 4:**
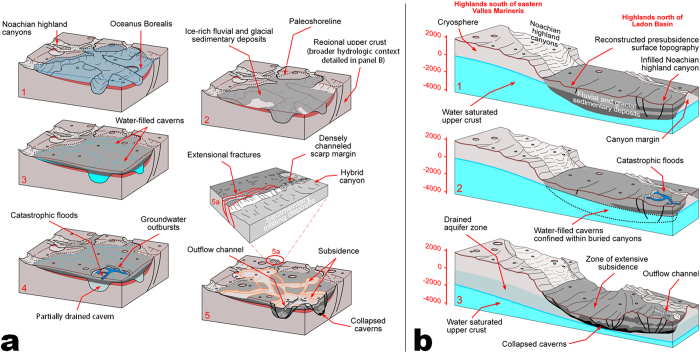
(**a**) Sketches depicting the inferred geologic evolution of a subsided highland plateau in southern circum-Chryse. (**1**) Submarine troughs become infilled with water-saturated sediments during the Late Noachian. (**2**) The sedimentary deposits freeze into permafrost upon the ocean’s retreat stage. (**3**) The troughs’ infilling deposits undergo melting to generate vast systems of interconnected water-filled caverns that extend to eastern Valles Marineris. (**4**) Groundwater outbursts lead catastrophic flooding. (**5**) Subsidence occurs over the evacuated caverns and hydrid canyons form close to the paleoshoreline elevation. (**5a**) View of a hybrid canyon. (**b**) Sketches depicting the inferred relationship between the eastern Valles Marineris, zones of subsidence and outflow channel formation in southern circum-Chryse. Ice/water-saturated sediments contained within buried troughs (**1**) undergo extensive melting and interconnect with a highly pressurized aquifer in eastern Valles Marineris (**2**). Extensive groundwater drainage along the trough interior deposits leads to outflow channel activity in southern circum-Chryse as well as to extensive subsidence over evacuated conduits (**3**). The pre- and post-subsidence surface elevations were constructed using MOLA elevation profiles and the topographic analyses described in the supplement. We produced the sketches using adobe illustrator.

We propose the following regional geologic reconstruction leading to the development of the OFCS aquifers: The vast fluvial, or glacial-glaciofluvial, canyon systems of southern Margaritifer Terra^4^,^23^ discharged enormous volumes of sediments into southern circum-Chryse, where lower portions of these canyons were completely buried and integrated into the regional upper crustal stratigraphy (Fig. 4a). Coeval fluvial activity through the huge Uzboi-Ladon-Morava channel system, which connects the Argyre basin to the northern plains, is thought to have discharged vast volumes of water generated by the melting of a Late Noachian south polar ice sheet^32^. This enormous fluvial system could have also contributed to regional sedimentation in southern circum-Chryse^2^,^33^. In the proposed geologic scenario the upper extent of regional sedimentation would have been controlled by the elevation of Oceanus Borealis (Fig. 3b, sketches 1 and 2 in Fig. 4a).

**Figure 5:**
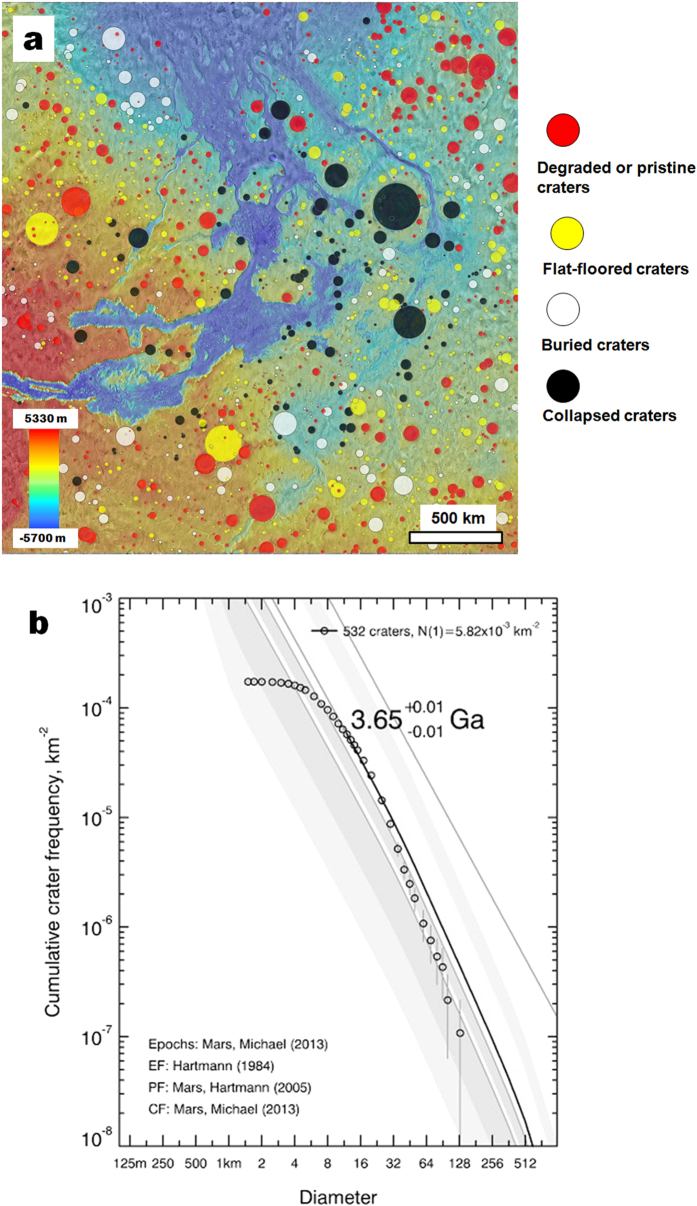
(**a**) Distribution of impact craters greater than 12 km in diameter. Measured impact craters include (1) collapsed craters, (2) buried craters, (3) flat-floored craters infilled up to their rims, and (4) degraded and pristine craters that retain significant topography. (**b**) Cumulative size-frequency distribution for all craters in study region. Calculated age includes craters with diameters larger than 12 km. Crater diameters were measured in ArcGIS software and cumulative size-frequency distributions were plotted using Craterstats2 software^59^. The Hartmann^60^ model production function and the Michael^59^ chronology function were used to calculate an overall age of 3.65 ± 0.01 Ga for the sedimentary wedge (i.e., Late Noachian^60^).

We infer a relatively short-lasting duration for the conditions allowing deep upland erosion connected with the formation of the proposed massive clastic sedimentary wedge based on previous investigations that propose a major spike in erosional and sedimentation rates occurring near the Noachian-Hesperian boundary^34^,^35^,^36^,^37^,^38^,^39^,^40^,^41^,^42^,^43^. This geologic stage likely lasted just a few million years^35^. To establish a relative chronology for the proposed stage of large-scale sedimentation, we mapped the distribution of (1) collapsed craters, (2) buried craters, (3) flat-floored impact craters infilled up to their rims, and (4) impact craters that retain significant topography (Fig. 5a). Our mapping of buried craters is based on the identification of quasi-circular depressions distributed throughout the regional highlands. These features are thought to have formed by compaction of sediments overlying buried impact craters^44^. Impact crater statistics yield a Late Noachian age of 3.65 ± 0.01 Ga (Fig. 5b), and point to a major spike in impact cratering rates during this time period. This spike was likely the result of a late phase of the Late Heavy Bombardment thought to have affected Mars up to 3.6 Ga^45^. Increased global erosional and depositional rates associated with an active surface hydrosphere^34^,^35^,^36^,^37^,^38^,^39^,^40^,^41^,^42^,^43^ during the Late Heavy Bombardment are consistent with impact-induced climate change as proposed by Segura *et al.*^46^.

The rapid groundwater evacuation required to generate the Late Hesperian catastrophic floods would have required groundwater migration via extraordinarily permeable structures, most likely including extensive systems of large interconnected caverns filled with water and/or unconsolidated fluidizable sediment^12^,^13^. We estimate that ~2.8 × 10^5^ km^3^ of groundwater and eroded or fluidized sediments were rapidly evacuated from the hypothesized caverns. This value was calculated as the total area of subsided highlands (~4 × 10^5^ km^2^) times an average negative relief of ~700 m [−1200 m minus −1900 m, respectively, elevation averages of undeformed highland plateaus and adjoining subsided terrains (Supplement)].

The distribution of the subsided valleys, here proposed to have been largely controlled by the distribution of the ancient buried troughs, indicates that the hypothesized caverns formed a direct connection between eastern Valles Marineris and a lower-lying equatorial belt of chaotic terrains located at the upstream portions of numerous outflow channels (dashed yellow line in Fig. 1a). These chaotic terrains occur within highland surfaces located within a narrow range of surface elevations (approx. ~500 m to −1000 m), which is consistent with groundwater outbursts produced by rapid release from an initially confined, underground body of water conforming to a potentiometric surface defined by the upper boundary of groundwater saturated crust in eastern Valles Marineris. Vast aquifers confined beneath a seal of ice-cemented permafrost are proposed to have existed within eastern Valles Marineris and nearby plateau regions during the Late Hesperian^47^,^48^,^49^. These aquifers are thought to have reached superlithostatic pressures as a consequence of ~3.5 to ~4.5 kilometers of hydraulic head generated due to the warping of regional upper crustal rocks by the Tharis uplift^19^.

Elaver Valles (Fig. 1a), located at an elevation of ~2000 m, is the highest outflow channel in eastern Valles Marineris. In contrast, the northern margin of the subsided terrain adjoining upper Ares Valles exhibits elevation ranges between approximately −1500 and −2500 m (Supplement). Moreover, elevation profiles from nearby undeformed highland surfaces show that the pre-subsidence topography was likely closer to approximately −1200 m, ranging between approximately −500 m and −1500 m (Supplement). These relief estimates provide a basis to quantify relevant hydraulic pressures. Prior to the catastrophic outflows, hydrostatic pressures within the confined, water-filled caverns could have attained h_w_g*ρ* between ~13 and 16.5 MPa (or 11 MPa, if corrected to pre-subsidence topography h_w_ (~3000 m)), where h_w_ is the relief in meters (~3500 m to ~4500 m), g is Martian surface gravity (3.711 m/s^2^), *ρ* is water density (1000 kg/m^3^). These estimates of regional groundwater pressurization approximate or exceed the upper estimate predicted to have caused outflow channel activity in circum-Chryse [10 MPa]^50^. The pressure may have been even greater if the groundwater was highly saline, saturated in CO_2_, or contained large volumes of fine-grained sediments. Following the groundwater outbursts, the hydraulic head within the previously confined cavernous water systems would have rapidly diminished due to drawdown of the regional water table, however, continued exsolution of CO_2_ could have maintained the outflow channel discharges.

Our proposed scenario implies that the sedimentary deposits that buried the troughs were highly porous and likely included large volumes of hydrated clays and perhaps glacal ice. As Oceanus Borealis receded, these water-saturated deposits would have progressively frozen into extensive areas of ice-rich permafrost (Fig. 4a, sketch 2), which remained stable for a few hundred million years.

Subsequent Late Hesperian groundwater eruptions and subsidence would have been controlled by the distribution of the buried troughs (Fig. 4a, sketches 3–5). Consequently, the water-filled caverns that produced the immense outflow channel discharges and related subsidence^12^,^13^ must have been excavated within the troughs’ permafrost or water-saturated infill. The generation and integration of these caverns have been attributed to localized permafrost melting by magmatic dikes in a way that large groundwater bodies were generated and confined by surrounding icy Late Noachian strata^12^,^13^,^21^, which likely contained significant volumes of less permeable phyllosilicates^23^. The integration of these caverns and their connection to a kilometers-deep aquifer system in Valles Marineris^47^,^48^,^49^ would have allowed for the role of superlithostatic pressures (Fig. 4b) in driving immense discharges to the southern Chryse basin. An alternative scenario might also involve the pressurization of meltwater beneath the Valles Marineris ice sheet^51^.

We find that Southern circum-Chryse had a unique geologic history that combined large-scale fluvial sedimentation along the margin of an ancient Late Noachian ocean; conduit development within the sedimentary materials infilling vast systems of buried troughs; and the development of superlithostatic pressures within the lower reaches of these conduits, perhaps driven by elevated potentiometric surfaces transferred from eastern Valles Marineris. High hydraulic pressure gradients across large regions of water-filled caverns permitted the rapid drainage of the immense groundwater systems. This region of the planet contains the largest outflow channel system on Mars, so our hypothesis, including the precursory erosional and sedimentation processes, centers on one of the most dramatic hydrogeologic stories in the Solar System.

On Mars there is evidence for the long-term retention of large groundwater volumes within buried impact craters^12^,^52^ and within thick upper crustal hydrated mineral deposits^53^. Clastic wedges containing large amounts of relic massive ice have been recognized within relatively young Earth deposits emplaced during recent glacial periods. These include glacial and glaciomarine sedimentary sequences^54^,^55^,^56^,^57^ produced during high sea-level stands^58^ that infilled and buried older valleys excavated by glacial and glaciofluvial erosion^54^,^55^,^56^,^57^. However, frequent warm climatic excursions on Earth imply that these ice masses can only remain stable over relatively short geologic periods. On the other hand, the colder climate and lower geothermal heat flow on Mars could have allowed Martian clastic wedges to retain vast volumes of ice during billions of years. This model and our new observations provide support for the Martian primeval ocean^24^,^25^,^26^,^27^,^28^ and link the formation of the vast outflow channel source region aquifers to the emplacement of thick water-rich upper crustal sedimentary deposits during a spike in global hydrologic activity^34^,^35^,^36^,^37^,^38^,^39^,^40^,^41^,^42^,^43^. The spike was perhaps triggered by impact-induced climate change^46^ during a late phase of the Late Heavy Bombardment^45^. If these extensive Late Noachian aquifer systems formed globally, then their development could have significantly depleted the surface hydrosphere, thereby playing a potentially significant role in triggering the abrupt transition into the colder and drier conditions that characterized most of the post-Noachian climatic regimes^1^. Thus, global Late Noachian sedimentary infill of buried canyons and craters might still retain much of the planet’s hydrosphere.

## Additional Information

**How to cite this article**: Rodriguez, J. A. P. *et al.* Martian outflow channels: How did their source aquifers form, and why did they drain so rapidly? *Sci. Rep.* **5**, 13404; doi: 10.1038/srep13404 (2015).

## Supplementary Material

Supplementary Information
