# Erratum: Martian outflow channels: How did their source aquifers form, and why did they drain so rapidly?

**DOI:** 10.1038/srep15092

**Published:** 2015-10-20

**Authors:** J. Alexis P. Rodriguez, Jeffrey S. Kargel, Victor R. Baker, Virginia C. Gulick, Daniel C. Berman, Alberto G. Fairén, Rogelio Linares, Mario Zarroca, Jianguo Yan, Hideaki Miyamoto, Natalie Glines

Scientific Reports 5: Article number: 13404; 10.1038/srep13404 published online: 09082015; updated: 10202015.

In the original version of this Article, the Abstract contained typographical errors.

“However, the restricted geographic location of the channels indicates that these conditions were not uniform Boundary.”

now reads:

“However, the restricted geographic location of the channels indicates that these conditions were not uniform.”

“Using more recent mission data, we argue that during the Late Noachian fluvial and glacial sediments were deposited into a clastic wedge within a paleo-basin located in the southern circum-Chryse region, which was then completely submerged under a primordial northern plains ocean.”

now reads:

“Using more recent mission data, we argue that during the Late Noachian fluvial and glacial sediments were deposited into a clastic wedge within a paleo-basin located in the southern circum-Chryse region, which at the time was completely submerged under a primordial northern plains ocean.”

In addition, there is a typographical error under ‘Results and interpretative synthesis’.

“The mean elevation of the Late Noachian ocean shoreline, which is the one of relevance to this study, has been estimated to be approximately −1680 m^24^.”

now reads:

“The mean elevation of the Late Noachian ocean shoreline, which is the one of relevance to this study, has been estimated to be approximately −1680 m^25^.”

These errors have now been corrected in both the PDF and HTML versions of the Article.

