# Supplementary material for: Martian outflow channels: How did their source aquifers form, and why did they drain so rapidly?
Source: Sci Rep. 2015 Sep 8;5:13404. doi: 10.1038/srep13404 (PMC4562069; doi:10.1038/srep13404)
Supplement: Supplementary Information [file srep13404-s1.doc]

**Supplement to:**

**Martian outflow channels: How did their source aquifers form, and why did they drain so rapidly?**

**J. Alexis P. Rodriguez^1,2^, Jeffrey S. Kargel^3^, Victor R. Baker^3^, Virginia C. Gulick^2,4^, Daniel C. Berman^1^, Alberto G. Fairén^5,6^, Rogelio Linares^7^, Mario Zarroca^7^, Jianguo Yan^8^, Hideaki Miyamoto^9^, and Natalie Glines^2,4^**

*^1^Planetary Science Institute, 1700 East Fort Lowell Road, Suite 106, Tucson, AZ 85719-2395, USA.*

*^2^NASA Ames Research Center, Mail Stop 239-20, Moffett Field, CA 94035, USA.*

*^3^Department of Hydrology & Water Resources, University of Arizona, Tucson, AZ 85721, USA.*

*^4^SETI Institute, 189 Bernardo Avenue, Mountain View, CA 94043.*

*^5^Centro de Astrobiología, M-108 km 4, 28850 Madrid, Spain.*

*^6^Department of Astronomy, Cornell University, Ithaca 14850 NY, USA.*

*^7^External Geodynamics and Hydrogeology Group, Department of Geology, Autonomous University of Barcelona , 08193 Bellaterra, Barcelona, Spain.*

*^8^State Key Laboratory of Information Engineering in Surveying, Mapping and Remote Sensing, Wuhan University, Wuhan, 430070, China.*

*^9^The University Museum, University of Tokyo, 113-0033, Japan.*


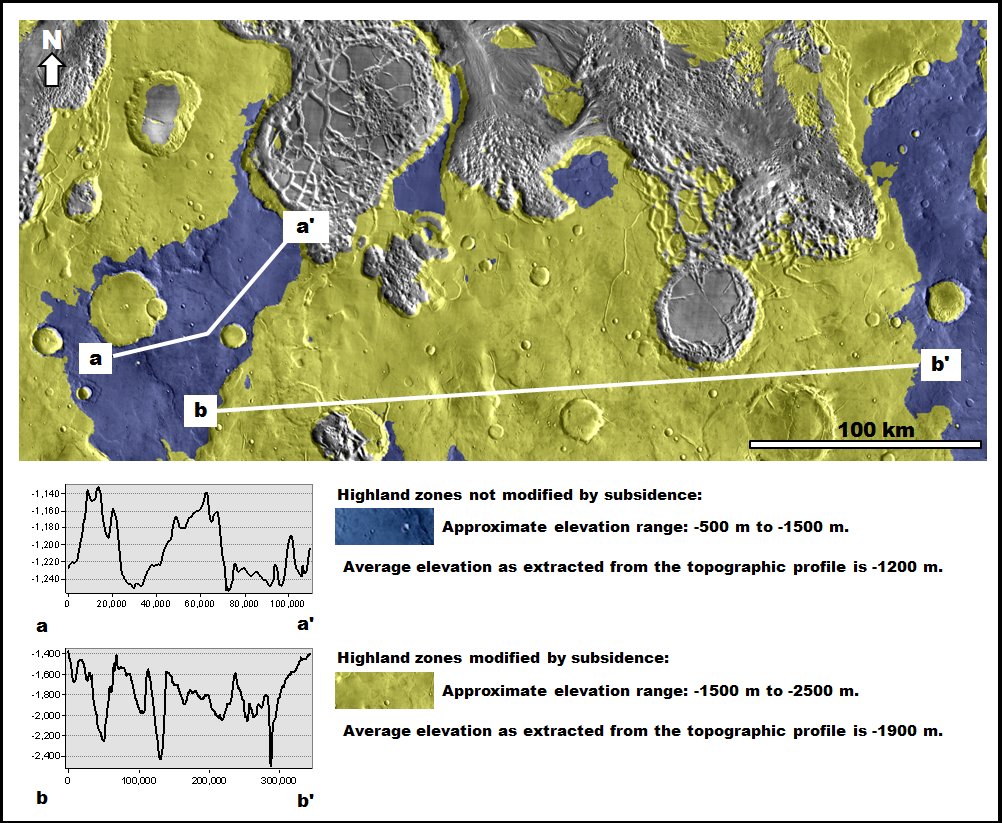


Shown are highland surfaces south of Hydaspis Chaos centered at 2°7'44"N, 26°26'45"W, which do not appear significantly warped (blue, profile a-a'), as well as those extensively modified by subsidence (yellow, profile b-b'). The map base is part of a [Mars Odyssey](http://mars.jpl.nasa.gov/odyssey/) THEMIS (Thermal Emission Imaging System) day-time infrared mosaic (512 pixels per degree). Graph lot axes are in meters. We produced this map figure using [Esri](http://en.wikipedia.org/wiki/Esri)'s ArcGIS [geographic information system](http://en.wikipedia.org/wiki/Geographic_information_system).
